# Pathological evaluation of a fluoropolymer-based drug-eluting stent in an arteriovenous graft outflow venous stenosis

**DOI:** 10.1016/j.jvscit.2024.101447

**Published:** 2024-02-08

**Authors:** Kotaro Suemitsu, Osamu Iida, Sho Torii, Yosuke Hata, Taku Toyoshima, Masaaki Izumi

**Affiliations:** a Division of Kidney and Dialysis, Department of Internal Medicine, Kansai Rosai Hospital, Amagasaki, Japan; b Cardiovascular Division, Osaka Police Hospital, Osaka, Japan; c Department of Cardiology, Tokai University School of Medicine, Isehara, Japan; d Cardiovascular Center, Kansai Rosai Hospital, Amagasaki, Japan

**Keywords:** Arteriovenous graft, Eluvia, Fluoropolymer-based drug-eluting stent, Hemodialysis, Vascular access

## Abstract

A fluoropolymer-based drug-eluting stent was implanted in an arteriovenous graft outflow venous stenosis. Two and a half years later, due to a local infection, the stent was removed surgically, and a pathological evaluation was conducted. The stent struts exhibited partial endothelial cell coverage, with the remaining surface predominantly covered by fibrin. Notably, there was no evidence of restenosis or aneurysmal change.

Arteriovenous fistulas represent the preferred option for vascular access (VA) in patients requiring hemodialysis. However, when arteriovenous fistulas are deemed infeasible, arteriovenous grafts (AVGs) serve as a valuable alternative. Nonetheless, AVGs are plagued by suboptimal primary patency rates, necessitating further intervention.^1^

The most frequent site of lesion formation in AVGs is the outflow vein. Traditionally, plain angioplasty has been used for managing these stenoses. However, the resulting secondary patency remains unsatisfactory for clinical practice. Recognizing this limitation, the Kidney Disease Outcomes Quality Initiative guidelines advocate for self-expanding stent grafts as the preferred approach for treating clinically significant graft–vein anastomotic stenosis in AVGs, surpassing the efficacy of plain angioplasty.^2^ Despite this recommendation, even stent graft placement falls short of achieving optimal outcomes, with a mere 51% target lesion primary patency rate at 6 months following intervention.^3^

Recently, fluoropolymer-based drug-eluting stents (FP-DESs) have emerged as a promising option for addressing dysfunctional AVGs, with encouraging reports of their efficacy.^4^^,^^5^ However, a crucial gap exists in our understanding of the long-term vascular response to FP-DES implantation in the outflow veins of AVGs, because no prior studies have investigated this aspect through pathological evaluation. This present work aims to bridge this gap by presenting a unique case with a pathological assessment of an FP-DES retrieved 2.5 years after its placement in an AVG outflow vein. This study seeks to provide a detailed pathological analysis of FP-DES effects on the outflow vein of AVGs, thereby shedding light on the safety and effectiveness of FP-DES implantation. The ethics committee approved the case report, and the patient provided written informed consent for the report of her case details and imaging studies.

## Case report

A 54-year-old woman with end-stage kidney disease due to diabetic kidney disease has been receiving hemodialysis since the age of 45. She was diagnosed with type 1 diabetes mellitus at the age of 10. She had no medical history of cardiovascular disease, including coronary artery or cerebrovascular disease. A 4.0- to 6.0-mm tapered expanded polytetrafluoroethylene loop graft (inflow, proximal radial artery; outflow, basilic vein) was implanted in her left forearm for VA at the initiation of dialysis.

**Fig 1:**
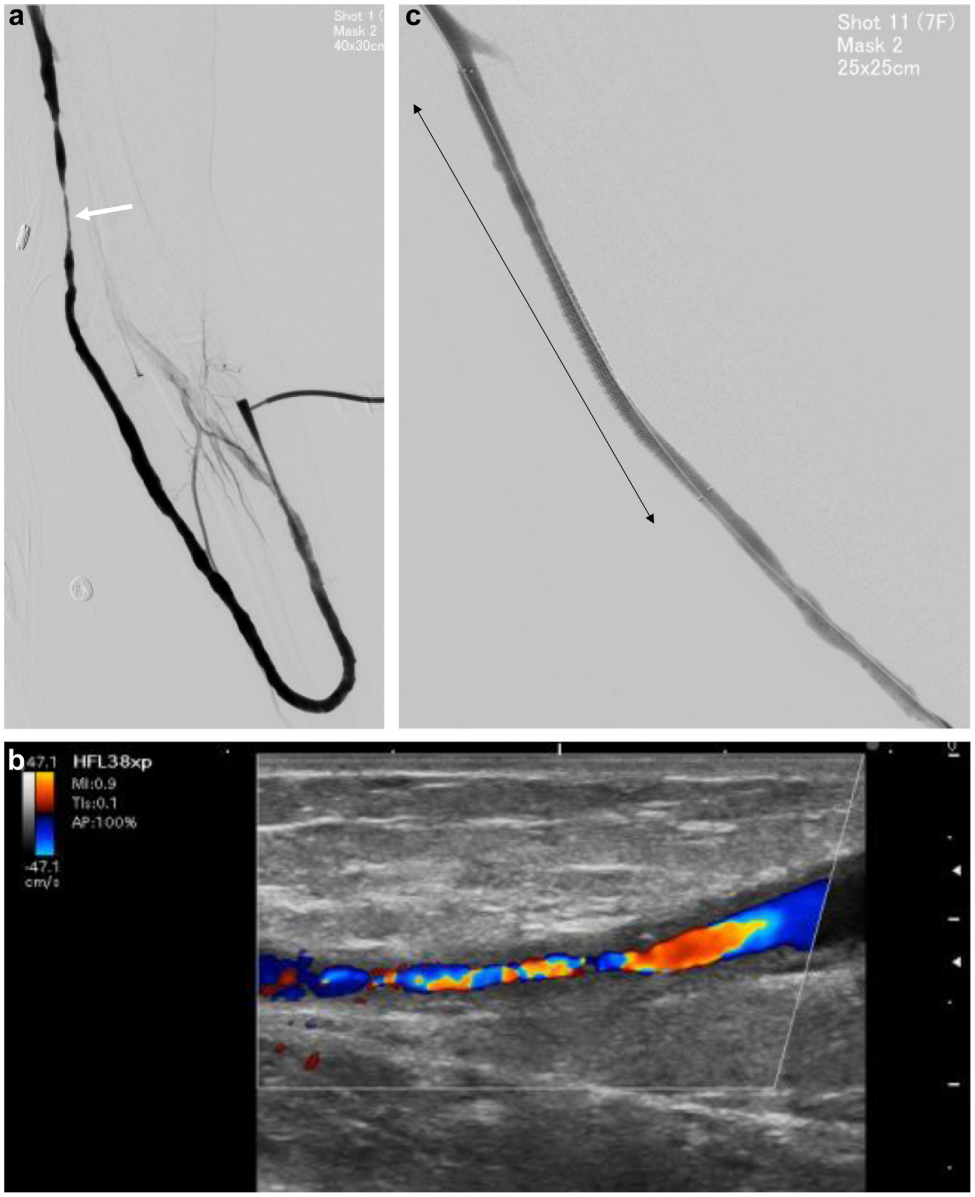
First fluoropolymer-based drug-eluting stent (FP-DES) implantation. **A,** Angiogram before first FP-DES implantation showing arteriovenous graft (AVG) with severe stenosis of the outflow vein (*white arrow*). **B,** Ultrasound image of the AVG outflow stenosis with intimal hyperplasia. **C,** Angiogram after FP-DES (Eluvia, 6.0 mm × 120 mm) was implanted on the outflow vein of the AVG (*black arrow*).

After 3 years, the patient developed outflow vein stenosis with reduced VA flow volume. Angioplasty with a non–drug-coated balloon was performed initially; however, restenosis recurred seven times during the next 3 years. Due to the increasingly rapid restenosis after each procedure, a 6.0- to 120-mm fluoropolymer-based paclitaxel (PTX)-eluting stent (Eluvia; Boston Scientific) was placed on the restenotic lesion (Fig 1). Administration of aspirin was then initiated.

**Fig 2:**
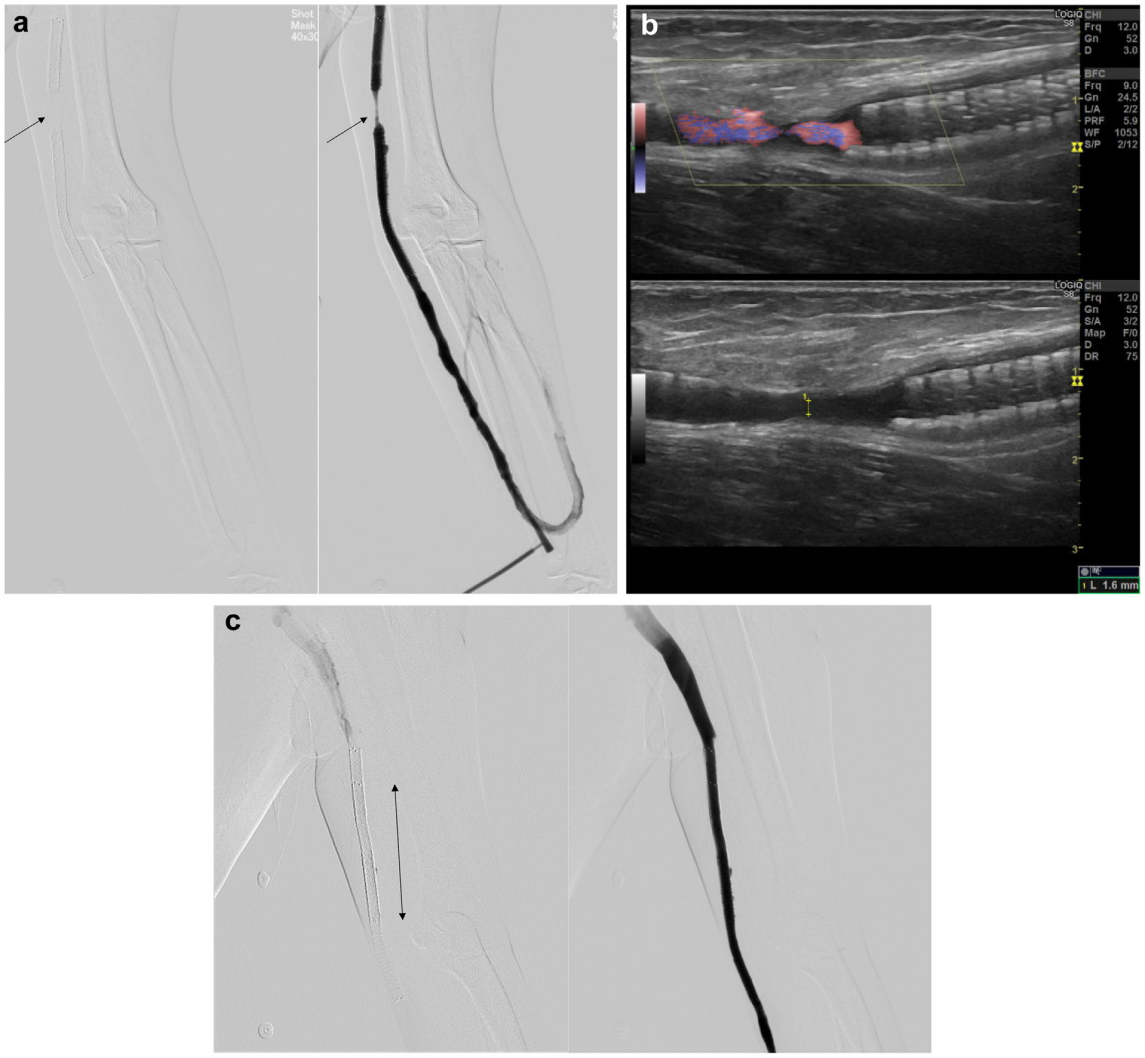
Four months after first fluoropolymer-based drug-eluting stent (FP-DES) implantation. **A,** Angiogram 4 months after FP-DES implantation An FP-DES fracture (*arrow*) occurred, and restenosis between the proximal and distal fracture developed. **B,** Ultrasound images 4 months after FP-DES implantation. An FP-DES fracture occurred, and restenosis between the proximal and distal fractures developed (1.6 mm). **C,** Angiogram after FP-DES implantation on the stent fracture. An FP-DES (Eluvia, 6.0 mm × 80 mm) was additionally placed on the restenotic lesion with the stent fracture (*black arrow*).

However, 4 months later, the FP-DES fractured, and restenosis occurred between the proximal and distal fracture segments (Fig 2, *A and B*). An additional 6.0- to 80-mm FP-DES was placed on the uncovered restenotic area (Fig 2, *C*).

**Fig 3:**
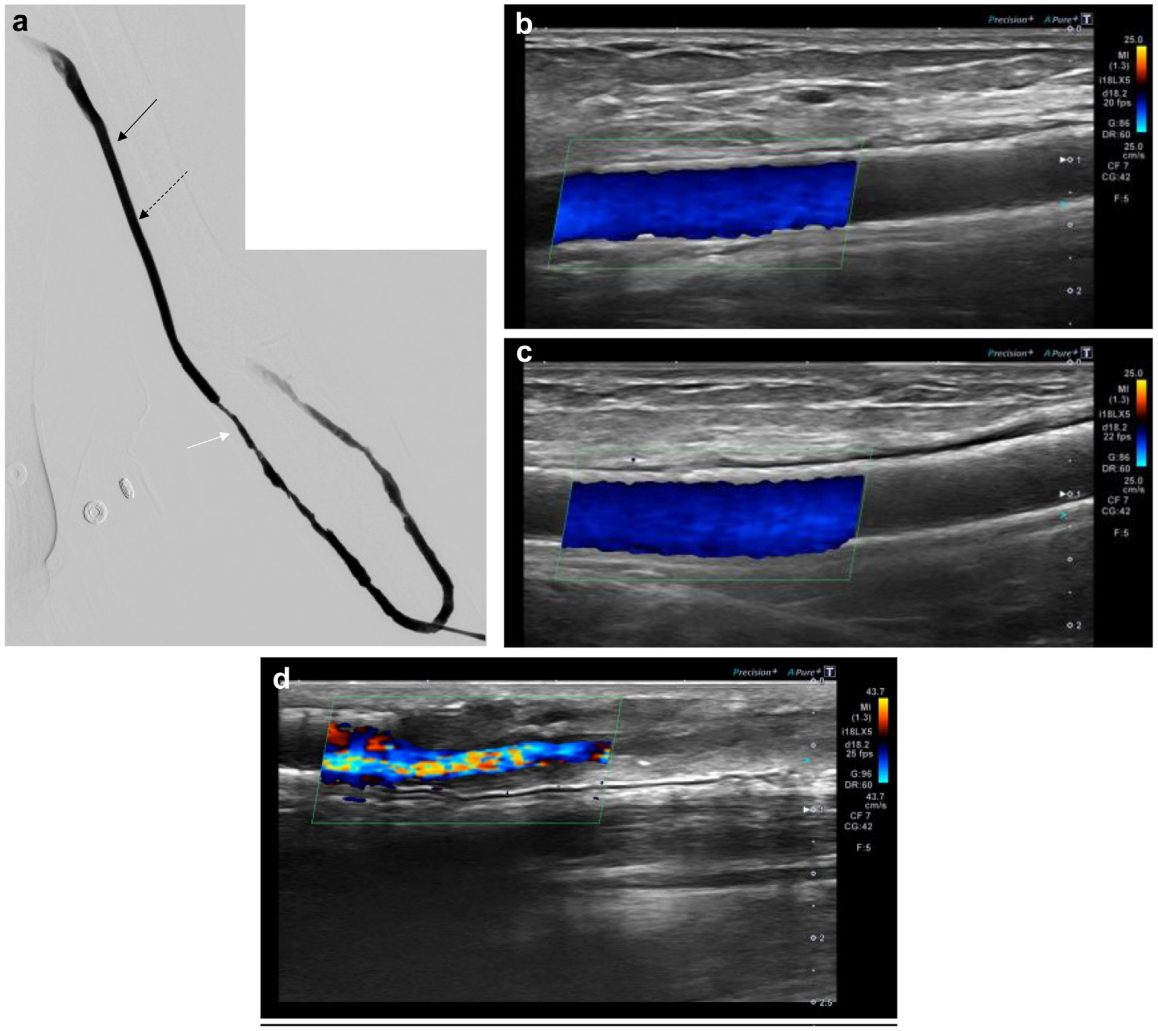
Puncture site stenosis. **A,** Angiogram of the puncture site stenosis showing no restenosis of the single fluoropolymer-based drug-eluting stent (FP-DES; *black arrow*) and overlapped FP-DES (*dotted arrows*). The arteriovenous graft (AVG) had diffuse stenosis on the puncture site (*white arrow*). **B,** Ultrasound image of the single FP-DES showing no restenosis in the FP-DES. **C,** Ultrasound image of the overlapped FP-DES showing no restenosis in the overlapped FP-DES. **D,** Ultrasound image of the venous puncture site showing stenosis at the venous puncture site.

Two years later, balloon angioplasty was performed at the graft's arterial and venous puncture site due to increased difficulty with cannulation. No stenosis was observed within the FP-DES during this procedure (Fig 3).

**Fig 4:**
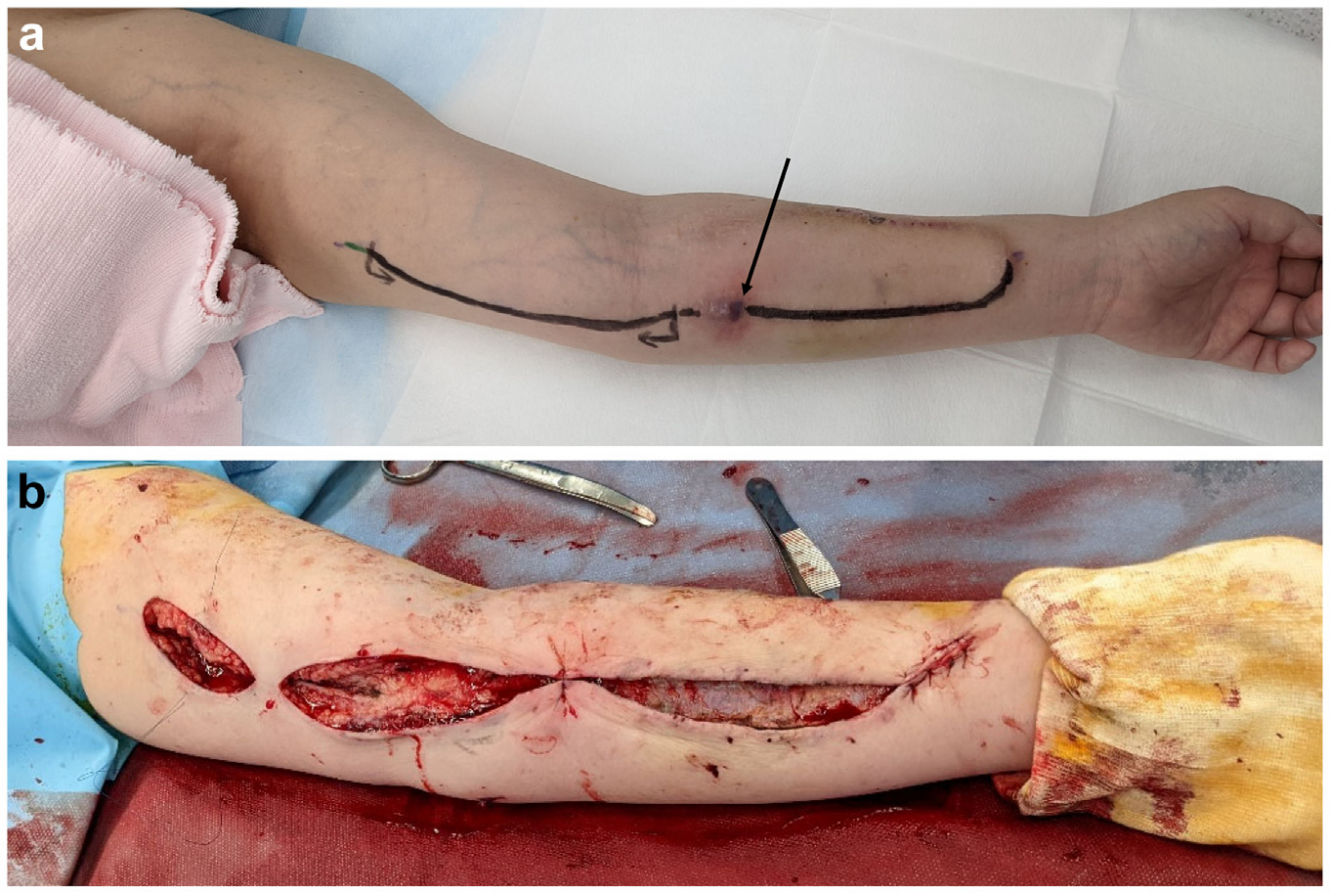
Graft infection. **A,** Graft infection of the venous puncture site. The arteriovenous graft (AVG) had a venous infection of the puncture site with erythema (*black arrow*). No erythema was found in the stent area on visual examination. **B,** Intraoperative findings. Partial graft removal was performed from the graft loop area to the fluoropolymer-based drug-eluting stent (FP-DES).

Four months after the angioplasty, an infection developed around the venous puncture site (Fig 4, *A*). Given the proximity of the stent and graft, it was suspected that the infection might have spread from the puncture site to the area near the end of the FP-DES. Partial graft removal was performed from the graft loop area to the FP-DES (Fig 4, *B*). The intraoperative findings revealed no pus around the stent, and *Staphylococcus epidermidis* was identified as the causative agent at the infection site. The patient was followed up for the next 15 months with no recurrence of infection.

## Pathological evaluation of Eluvia stent

**Fig 5:**
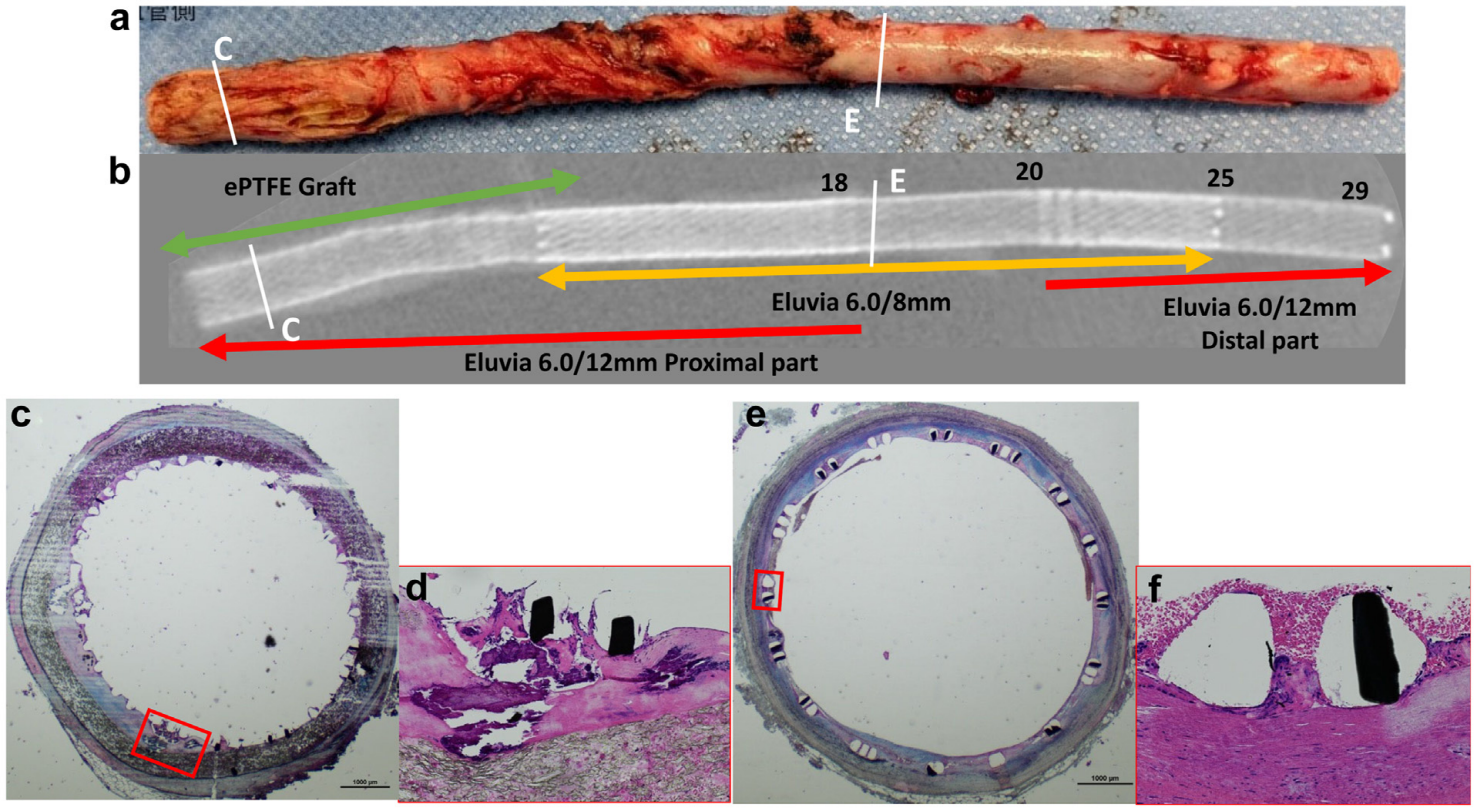
Pathological findings of the fluoropolymer-based drug-eluting stent (FP-DES). Gross (**A**) and radiographic (**B**) images of the removed portion. Histopathological sections of the overlapped part with expanded polytetrafluoroethylene and FP-DES (**C**; stained with Movat pentachrome stain) demonstrate vessel patency and fibrin deposition around the stent struts without any endothelial coverage. **D,** Note the deposition of the calcification in the fibrin in the high-power image of the *red-boxed area* in **C**. **E,** The FP-DES implanted lesion to the arteriovenous shunt vein stained with Movat pentachrome stain. **F,** One portion of the stented struts was covered by neointima; however, the coverage of the rest remained rare, with only postmortem red blood cells around the struts in the high-power image of the *red-boxed area* in **E**.

Pathological evaluation of the overlapped lesions involving the expanded polytetrafluoroethylene graft and FP-DES (Fig 2, *green arrow*) and the single FP-DES implantation site revealed patent lesions (Fig 5). Notably, the degree of inflammation in the stented lesion was mild, suggesting that these areas might not be the primary source of infection, particularly in the absence of signs such as mycotic aneurysm. Interestingly, even 2.5 years after implantation, the FP-DES struts were predominantly surrounded by fibrin with only partial endothelial cell coverage. Furthermore, mild calcium deposition was observed within the fibrin at the overlapped segment.

## Discussion

Encountering a case of AVG outflow vein stenosis treated with FP-DES, we conducted a pathological evaluation of the FP-DES 2.5 years later. Several studies of treating peripheral arterial disease have shown that long-term PTX release after FP-DES placement effectively reduces the target lesion revascularization rate.^6^ However, other reports have raised clinical concerns regarding aneurysmal changes.^7^ Therefore, understanding the vascular changes after FP-DES implantation for dysfunctional AVGs is of significant clinical interest.

Outflow vein restenotic lesions following polytetrafluoroethylene dialysis graft placement have generally been characterized by the presence of smooth muscle cells, myofibroblasts, and angiogenesis.^8^ Given PTX's known mechanism of inhibiting smooth muscle proliferation, FP-DESs were predicted to be effective in this regard. Notably, the pathological findings in the present case also confirmed the absence of restenosis in the target lesion even 2.5 years after FP-DES implantation.

PTX-DESs used in coronary artery bypass grafting with saphenous vein grafts demonstrate specific features compared with bare metal stents, including fibrin deposition around the struts, a higher percentage of uncoated struts, and decreased neointima formation.^9^ Yamamoto et al^10^ evaluated the efficacy of bare metal stents in graft outflow stenosis based on lesion morphology using ultrasound. They found them effective only for vascular construction types without neointimal hyperplasia. Their effectiveness was limited in the neointimal proliferation and mixed types. In contrast, FP-DESs with the ability to prevent neointima proliferation is potentially effective for any lesion type of AVG outflow stenosis.

Although offering benefits, the use of FP-DESs also presents some concerns. Delayed neointimal coverage due to long-lasting PTX release raises concerns about late thrombotic occlusion, potentially necessitating continued antiplatelet therapy. However, balancing the risk of bleeding with the need for antiplatelet therapy in this context remains undetermined, and the optimal type, dose, and duration of antiplatelet therapy requiring further investigation.^11^ Aneurysmal degeneration after FP-DES implantation is another concern, with a reported occurrence of 16.8% in peripheral arterial disease.^12^ Although no clinical or pathological evidence of this was observed in our case, careful postimplantation monitoring remains crucial.

## Conclusions

Pathological evaluation 2.5 years after FP-DES implantation for outflow vein stenosis in AVGs revealed partial endothelial cell coverage on the stent struts, with fibrin as the predominant surrounding material. Notably, no evidence of restenosis or aneurysmal change was observed.

## Disclosures

K.S. received honoraria from Medtronic Japan and Boston Scientific Japan. O.I. is a consultant who received honoraria from Medtronic Japan. S.T. received research grants from Abbot Vascular Japan, Boston Scientific Japan, and Medtronic, and received honoraria from Boston Scientific Japan. Y.H., T.T., and M.I. have no conflicts of interest.

## References

[1] Miller P.E., Carlton D., Deierhoi M.H., Redden D.T., Allon M. (2000). Natural history of arteriovenous grafts in hemodialysis patients. Am J Kidney Dis.

[2] Lok C.E., Huber T.S., Lee T., National Kidney Foundation (2020). KDOQI clinical practice guideline for vascular access: 2019 update. Am J Kidney Dis.

[3] Haskal Z.J., Trerotola S., Dolmatch B. (2010). Stent graft versus balloon angioplasty for failing dialysis-access grafts. N Engl J Med.

[4] Katsanos K., Ho P., Tang T.Y. (2023). Polymer-coated paclitaxel-eluting stents for the treatment of stenosed native arteriovenous fistulas: long-term results from the ELUDIA study. J Vasc Access.

[5] Matsuoka Y., Iida O., Suemitsu K., Oka K., Ota N., Izumi M. (2021 Apr 20). Use of a fluoropolymer-based paclitaxel-eluting stent for arteriovenous graft outflow vein stenosis in hemodialysis patients. J Vasc Surg Cases Innov Tech.

[6] Müller-Hülsbeck S., Benko A., Soga Y. (2021). Two-year efficacy and safety results from the IMPERIAL randomized study of the eluvia polymer-coated drug-eluting stent and the zilver PTX polymer-free drug-coated stent. Cardiovasc Intervent Radiol.

[7] Haraguchi T., Takahara M., Iida O. (2023). Impact of postprocedural minimum lumen area on clinical outcome after femoropopliteal drug-eluting stent implantation. Vasc Med.

[8] Roy-Chaudhury P., Kelly B.S., Miller M.A. (2001). Venous neointimal hyperplasia in polytetrafluoroethylene dialysis grafts. Kidney Int.

[9] Yazdani S.K., Farb A., Nakano M. (2012). Pathology of drug-eluting versus bare-metal stents in saphenous vein bypass graft lesions. JACC Cardiovasc Interv.

[10] Yamamoto Y., Nakamura J., Nakayama Y., Hino H., Kobayashi H., Sugiura T. (2012). Relationship between the outcomes of stent placement and the properties of arteriovenous graft outflow vein stenotic lesions. J Vasc Access.

[11] Hiremath S., Holden R.M., Fergusson D., Zimmerman D.L. (2009). Antiplatelet medications in hemodialysis patients: a systematic review of bleeding rates. Clin J Am Soc Nephrol.

[12] Iida O., Takahara M., Soga Y. (2022). CAPSICUM investigators. 1-Year outcomes of fluoropolymer-based drug-eluting stent in femoropopliteal practice: predictors of restenosis and aneurysmal degeneration. JACC Cardiovasc Interv.

